# The Human Microbiota and Obesity: A Literature Systematic Review of In Vivo Models and Technical Approaches

**DOI:** 10.3390/ijms19123827

**Published:** 2018-11-30

**Authors:** Lucrecia Carrera-Quintanar, Daniel Ortuño-Sahagún, Noel N. Franco-Arroyo, Juan M. Viveros-Paredes, Adelaida S. Zepeda-Morales, Rocio I. Lopez-Roa

**Affiliations:** 1 Laboratorio de Ciencias de los Alimentos, Departamento de Reproducción Humana, Crecimiento y Desarrollo Infantil, Universidad de Guadalajara, CUCS, Guadalajara Jalisco 45180, Mexico; lucrecia.carrera@gmail.com; 2 Laboratorio de Neuroinmunobiología Molecular, Instituto de Investigación en Ciencias Biomédicas (IICB) CUCS, Universidad de Guadalajara, Guadalajara Jalisco 45180, Mexico; daniel.ortuno.sahagun@gmail.com; 3 Laboratorio de Investigación y Desarrollo Farmacéutico, Universidad de Guadalajara, CUCEI, Guadalajara Jalisco 44430, Mexico; noel_n_franco@hotmail.com (N.N.F.-A.); jvivero99@hotmail.com (J.M.V.-P.); adelaida.zepeda@academicos.udg.mx (A.S.Z.-M.)

**Keywords:** microbiota, obesity, human, animal model, inflammatory disease

## Abstract

Obesity is a noncommunicable disease that affects a considerable part of humanity. Recently, it has been recognized that gut microbiota constitutes a fundamental factor in the triggering and development of a large number of pathologies, among which obesity is one of the most related to the processes of dysbiosis. In this review, different animal model approaches, methodologies, and genome scale metabolic databases were revisited to study the gut microbiota and its relationship with metabolic disease. As a data source, PubMed for English-language published material from 1 January 2013, to 22 August 2018, were screened. Some previous studies were included if they were considered classics or highly relevant. Studies that included innovative technical approaches or different in vivo or in vitro models for the study of the relationship between gut microbiota and obesity were selected after a 16-different-keyword exhaustive search. A clear panorama of the current available options for the study of microbiota’s influence on obesity, both for animal model election and technical approaches, is presented to the researcher. All the knowledge generated from the study of the microbiota opens the possibility of considering fecal transplantation as a relevant therapeutic alternative for obesity and other metabolic disease treatment.

## 1. Introduction

Obesity is one of the most alarming diseases of the 21st century. The World Health Organization (WHO) has reported that more than 1.9 billion adults around the world are overweight and nearly one-third of such individuals are obese [1]. In the last years, much of the research has focused on understanding the pathogenesis of obesity and its development, as well as looking for methods and alternatives to solve such a worldwide problem [2].

Gut microbiota is the set of bacteria, fungi, and protozoans that colonizes the digestive tract in a symbiotic consortium that has been recognized as a complex system that contributes to the physiological processes of the host. Recently, gut microbiota has been described as a fundamental factor in the triggering and development of a large number of pathologies, among which obesity is one of the most highly related to the processes of dysbiosis, including the relationship with the diversity and quantity of phyla such as Firmicutes and Bacteroidetes [3].

Unfortunately, studying obesity and microbiota directly in humans is extremely difficult due to challenges of controlling variables in study subjects such as diet, medication, and genotype, as well as obstacles regarding the obtainment of samples and measurements. Thus, the first phases of research usually rely on in vitro or nonhuman in vivo models, in order to acquire a mechanistic understanding of the microbiome and obesity relationship. This review focuses on the main animal models and methods used to study human gut microbiome characteristics and their impact on obesity and metabolic diseases.

## 2. Animal Models as Tools to Study the Human Gut Microbiota

The microbiota is implicated in diverse aspects of human health, including gut and immune development, energy balance, resistance to infections, and the processing of foreign compounds. Many of these associations have been made through studies in animal models in which the presence or composition of the microbiota could be manipulated. Microbiota contributes to host phenotypes. For example, many diseases are frequently related to perturbations in the microbial community, and its modification, for example by nutraceutics as phytochemicals, can be prophylactic for the prevention or treatment of obesity and inflammatory diseases [4]. The utility of model systems of the microbiome depends on how well features observed in the model represent the native (human) system. Studies of the human gut microbiome in animal models allow microbial communities to be evaluated in a standardized and replicable host context in which key complicating features of the human host can be controlled (Table 1) [5].

### 2.1. Non-Mammalian Models of the Human Microbiome

Simple multicellular organisms provide an opportunity to identify evolutionary conserved features of the host–microbe relationship with a reduced complexity compared to mammals. For instance, these relationships may be represented by less than 50 species per host in simple multicellular organisms [6].

#### 2.1.1. *Hydra*

The *Hydra* belongs to the phylum Cnidaria and is considered a phylogenetically basal model with a simple body, a limited number of cells, nervous and immune systems, and a tube-like body with spontaneous contraction that resembles a mammalian intestine [7]. Disturbances in epithelial homeostasis of this model lead to changes in the microbiome consortium, providing the opportunity to study such phenomena that are similar to mammals [8]. It also has preserved ancestral genes shared with humans that were lost in *Drosophila* and *Caenorhabditis* [9] and is able to secrete antibacterial peptides to shape its microbiome [10]. The benefits of using *Hydra* include the possibility of performing germ-free, gnotobiotic, or bacteria-specific models, its transparent body, its fast life cycle, and its easy cultivation, all of which make it a good model for the study of metaorganisms and interactions with hosting bacteria. The main bacteria present in *Hydra* can be cultured and Deines et al. proposed an in vivo and in vitro system of studying the interactions within the microbiome in *Hydra*, which provided insights into how the host affects intermicrobe interactions and vice versa [11].

#### 2.1.2. Honeybee

The microbiome in the gut of vertebrates is represented by only a few phyla, but there is considerable intraspecies variation [6]. In many diseases such as obesity or inflammatory bowel disease, a wide variation of bacteria strains is detected, impacting the specific adaptive functions [12]. Therefore, invertebrates are an excellent option for studying intraspecies diversity in the gut using metagenomic sequencing to obtain a thorough analysis of the microbiota. The honeybee (*Apis mellifera*) hosts eight bacterial species in its gut, with *Snodgrassella alvi* and *Gilliamella apicola* being the most abundant, that are transmitted between individuals through social interactions [13]. However, similar to humans, the core gut microbiota has a set of characteristics shared among most microbiomes and usually refers to genes or the metabolic abilities of the honeybee, which include a great amount of intraspecies diversity that affects metabolism and immune functions [14].

How the honeybee gut maintains its intraspecies diversity remains unknown, and the distribution of the microbe diversity within a honeybee colony has not been deeply studied. A simple explanation for the observed stable coexistence is that despite bacterial strains from the same species, they occupy distinct functional groups in accordance with nutrients or different types of diets. In that respect, data from metagenome studies in honeybees have shown different sublineages being present within the *Lactobacillus apis* group, which suggests that different evolutionary lineages with different functional roles (e.g., specific polysaccharide utilization) compose the intraspecies diversity of the gut microbiota [15].

#### 2.1.3. Zebra Fish

The zebrafish (*Danio rerio*) is an omnivorous freshwater teleost with many shared features of its digestive tract with mammals and is described as a model for studying microbiota interactions and their impact on host nutrient absorption, metabolism, the immune system, and intestinal epithelial functions [16]. The zebrafish is an ideal model for the study of microbiota due to the possibility of sampling the whole environment and the use of the same diet throughout the life of the host, besides it having a transparent body, good fecundity, and the ease to maintain germ-free environments with hundreds of individuals [17]. Preliminary results provided by 16S rRNA sequencing of zebrafish indicate that its gut microbiota is primarily colonized by the Proteobacteria phylum at all stages of its life, but also by Firmicutes during the larva stage and *Fusobacteria* during adult ages [18].

Dietary fat consumption is associated with microbiome changes, influencing both gut and environmental microbial ecologies [19], but little is known regarding the impact of high caloric and long-term diet *alteration* outside the mammal models. Using a zebrafish model with a constant diet, Wong et al. reported how the gut microbial communities changed through age for both the microbes colonizing the intestine and the ones present in the environment. Also, the amount of fat consumed led to different age-specific effects on the gut consortium [20]. Valenzuela et al. have shown the capability of specific human gut microbiota such as *Lactobacillus acidophilus*, *Bifidobacterium. adolescentis*, and *Clostridium difficile* to colonize zebrafish larvae with a persistence of only a few days [21]. Additional studies are required to generate a zebrafish model for research with functional human microbiota. 

### 2.2. Mammalian Models of the Human Microbiome

#### 2.2.1. Germ-Free (GF) Mice

Microbiota contribute to host phenotypes. For example, many diseases are frequently associated with perturbations in the microbial community. A common method to study the interactions between diet, microbiota, and the underlying mechanisms of the development of obesity is the generation of germ-free animals (most commonly mice) devoid of microbes that are resistant to body weight increases induced by diet compared to conventional animals. Several lines of evidence have shown a relationship between metabolism in GF mouse models and their microbiota [22].

In 2004, Bäckhed et al. established that the energy yield and storage from the diet in hosts are affected by the microbiota. They observed that colonization of GF C57BL/6 with microbiota from the caecum of normally raised mice resulted in reduced food consumption but triggered the development of insulin resistance and body fat increases within two weeks. In addition, they identified that the suppression of fasting induced adipose factor (FIAF), a protein essential for the induction of disposal of triacylglycerols in adipocytes through microbiota signals [23]. Polysaccharides that cannot be digested by mammalian enzymes are transformed into bioavailable sugars by gut microbiota, which thereby regulate the immune system and nutrient absorption [24]. Interesting results obtained by Wang et al. in a model using conventional and GF mice showed that the microbiota also regulates body fat accumulation through the induction of circadian transcription factor NFIL3 expression, a mechanism that explains the impact of circadian disruptions on the development of metabolic disease [25].

Although gnotobiotic technologies for GF animals have been explored since the 1950s [26], the use of GF mice remains a limited approach by investigators. The use of antibiotics to alter microbiome composition to nearly deplete all the microbes is an economically feasible approach. Protocols using cocktails of antibiotics for approximately one month are widely used alternatives and allow the study of the pathogenesis in a variety of models where the microbiota modulates host functions [27].

GF mice in gnotobiotic models can be colonized by single, specific types of bacteria to study mono-associated situations or can be colonized by two or three specific bacterial types to generate a simplified microbiota. GF mice can also be transplanted with culture collections or human fecal microbiota to investigate functions of complex human-derived microbiota. For example, research using GF mice transplanted with human microbiota has shown that cross-feeding activities of *Akkermansia muciniphila* establish the mucus-degrading ability of this bacterium and its capacity to produce short chain fatty acids (SCFAs), which stimulate microbiota interactions and host modification that protects intestinal integrity from pathogenic bacteria [27]. The health-promoting effects of probiotics have also been tested with GF mice colonized with human gut-derived species. Sugahara et al. elucidated the molecular elements for the beneficial effect of *Bifidobacterium longum* BB536 interactions with the microbial consortium on the gut luminal metabolism [28]. It is important to consider whether the physiology of GF mice is altered compared to that of conventional mice due to an aseptic environment with differences in immune and intestinal functions, which then affects the pathogenesis of metabolic diseases [29].

#### 2.2.2. Rat

The rat was the first mammal to be employed as an animal model for scientific purposes. The bacterial phyla in a healthy rat gut are similar to those in human and mouse guts, hosting Firmicutes, Bacteroidetes, Proteobacteria, and Actinobacteria, but the differences compared to those of humans should be considered. For instance, the large intestine of the rat presents a more complex environment, with rat feces having 2–3 times the bacterial diversity compared to human feces [30]. *Lactobacillus* species represent 10%–15% of the rat microbiome: The rat microbiome is rich in uncharacterized anaerobic fusiform-shaped bacteria and segmented filamentous bacteria [31]. *Faecalibacterium* is a bacteria present in the healthy human intestine, but which displays a very low abundance in the healthy rat intestine [32]. 

Despite these differences and other disadvantages such as elevated costs and more difficultly in their manipulation compared to mice, rats are good models for specific pathogen-free (SPF) experiments because their gut microbiome can be established under conventional or SPF conditions after the introduction of certain controlled environments [33]: They have been used to analyze the influence of antimicrobial treatment on intestinal microbiota [34], to generate “human microbiota-associated” animals [35], to test the effect of certain probiotics [36], and to evaluate the relation of microbiota characteristics in obesity [37]. On the other hand, host–gut microbial interactions may vary between different rat strains, which represents a difficulty for their use in studies and therefore the impact of diet on the makeup of gut microbiota differs slightly between studies [38].

#### 2.2.3. Pigs

The use of rodent animal models is useful for many objectives, providing models with low costs of breeding, feeding, and handling, but their use has some disadvantages, such as differences compared to humans with respect to physiology and metabolism. For instance, rodents feed mainly as granivore animals in contrast to humans, have faster digestive passage and less fiber digestion, and include fermentation processes in the large caecum. Regarding the gut microbiota, the presence of important genera such as *Lactobacillus* and *Bifidobacterium* spp. is different in abundance between rodents and humans [39].

Compared to humans, the pig presents similarities in anatomical structure and gastrointestinal tract functions, metabolism and diet requirements, and importantly a similar microbiome in the gastrointestinal tract (Firmicutes, Bacteroidetes) [40]. Fermentation processes take place in the colon with similar microbiota composition in both pigs and humans, but pigs exhibit a significant level of caecal fermentation and obtain almost one-third of their energy requirements from SCFA generated by the microbiome [41], compared to humans, who obtain less than 10% of their energy requirements through such a mechanism [42]. As expected, the gastrointestinal bacterial community of pigs responds to changes in diet. For example, in a study using moderate dietary protein restriction on the microbiota of finishing pigs, Fan et al. reported that this diet altered the bacterial community through a beneficial bacterial profile in the ileum and colon, as well as improved the gut barrier function [43].

In addition, compared to other animal models, pig models allow for deeper sampling of the gastrointestinal tract and easier induction of diseases under a more controlled diet intake compared to humans. Also, adipocyte size and adipose tissue distribution in pigs are similar to those in humans and the sedentary behavior and fattening of pigs resemble human characteristics [44]. Using an obese-pig model, Heinritz et al. assessed the impact of high-fat diets or high-fiber consumption. They observed both quantitative and qualitative differences in microbial composition, levels of SCFA, and biochemical parameters like glutamic oxaloacetic transaminase, serum glutamic pyruvic transaminase, glucose, and the low-density lipoprotein/high-density lipoprotein ratio [44].

A summary of the in vivo models and their previously mentioned possibilities for the microbiota study is shown in Table 2.

## 3. Methodologies for the Study of Microbiota

Animal testing is highly useful in analyzing the effect of diet on gut microbes. However, the ethical issues and costs are disadvantages and may restrict their application. An alternative option to this is the use of in vitro intestinal models, which have advantages like reproducible experimentation and standardized conditions. However, these models have limitations in experimental duration, quantity of substrate supply, and a dependence on the inoculation density. In contrast, models using continuous culture systems allow the control of parameters like dilutions, retention time, pH, temperature, substrate replenishment, and toxic waste removal to maintain optimal growth conditions and represent excellent options for use in experimental studies on microbial composition and activity [49].

Regarding continuous in vitro models, the novel Polyfermentor Intestinal Model (PolyFermS) allows for consecutive testing of the impact of different experimental conditions on the same microbiota complex. Poeker et al. used PolyFermS with a first-stage reactor and inocula from two adult proximal colon microbiota and five second-stage reactors to evaluate different conditions and to test the effect of fermentable dietary fibers on the microbiome metabolic functions and composition [50]. They found that the response to such prebiotics were individual-dependent [50].

### 3.1. Culturomics and Matrix-Assisted Laser Desorption/Ionization–Time of Flight (MALDI–TOF)

Culture-based approaches were used during the 1970s at the start of efforts to characterize the human gut microbiota [51]. However, culture techniques were considered fastidious processes for many reasons: (a) The lack of a rapid identification method; (b) many species being unable to grow or be isolated using classic culture techniques; and (c) the high cost of this approach for such objectives [52]. On the other hand, molecular techniques such as metagenomics advanced the research of the human microbiome, but a problem was that some of the DNA sequences obtained could not be attributed to any known bacteria species [53]. 

Culturomics aims to solve such problems and to fill the gaps in knowledge regarding the human microbiome that are required for researching the microbiota and its relation to human health by growing microorganisms in pure cultures [54]. Culturomics consists of the application of highly controlled and specific culturing to the study of the human microbiota, employing matrix-assisted laser desorption/ionization–time of flight or 16s rRNA amplification and sequencing to identify currently unidentified colonies with the advantages of speed, inexpensiveness, and the possibility of performing differentiation at a species level or even at a strain level [55]. Isolated pure colonies are required to generate MALDI–TOF samples, which are prepared in a matrix mix of organic compounds with the capacity to absorb energy. The matrix crystallizes as a result of the drying procedures, and the crystallized sample is placed onto an instrument with a laser beam for desorption and ionization of the sample components. The ionized peptides travel through a vacuum tube and are sifted using fixed potential depending on their mass-to-charge ratio (*m*/*z*), detected by mass TOF analyzers, and finally are compared with database references [56].

### 3.2. Metagenomics

Using 16s rRNA gene amplicon sequencing and operational taxonomic unit (OTU) clustering with a high-match cutoff for sequence identity of 97%, the gut microbiota diversity can be described at the species level [57,58]. However, such classification can be arbitrary. Human gut microbiota studies have assigned 98% of 16S rRNA sequences to four main phyla: Firmicutes (64%), Bacteroidetes (23%), Proteobacteria (8%), and Actinobacteria (3%) [59]. However, a simple phyla composition is not enough to describe a relationship between microbiota and disease, and recent evidence links obesity not only to certain bacteria species, but to strain levels with marked genetic variation [60]. The similarity in the 16S rRNA sequence between the members of an intraspecies group, determined by this approach, is deficient to go deep into strain characterization, making the genome-wide approaches better for that purpose [6].

Metagenomics, originally used to study complex ecosystems of the environment [61], is a proposed tool to characterize the “uncultivable” members of the microbiota [62]. This approach analyzes the functional-gene composition and makes comparisons using databases such as the Ribosomal Database Project (RDP) [63] and the Ribosomal RNA Database Project, named SILVA [64], bringing a deeper description than those provided by approaches based on a single gene.

Whole-community shotgun sequencing is a metagenomic technique where DNA is obtained from an environmental sample and shotgun-sequenced, and the resulting DNA sequence data are either pieced together using assembly algorithms or analyzed unassembled to monitor whole-community functional capabilities [65]. The primary advantage of this approach is that it allows for simultaneous monitoring of diverse microorganisms, not only the species that grow readily in the laboratory. In addition, since there is no polymerase chain reaction (PCR) step, it is free from some of the biases introduced by DNA amplification [66] (Table 3). 

Thus, metagenomics is able to determine the abundance of specific functional genes in the corresponding database that can be derived by protein sequences or protein families [72]. From there, the challenge is not only to describe, but to infer and test causal relationships between specific microbes and pathologies. To achieve such objectives, the scientific community has developed through the years multiple microbiome databases. These databases provide not only information regarding the phylum or abundance of the microbiota, but also information related to microbial metabolism by identifying genes involved in metabolic functions that regulate the biotransformation, availability, and absorption of nutrients, and thus affect host physiology [73]. By this way, modeling can be grouped into two types of approaches. First, the genome-scale metabolic models (GEMs) are based on reconstruction of the metabolic networks of specific organisms (even plants or mammals) analyzed using simulation frameworks and provide the opportunity to integrate data such as transcriptomic and proteomic data using the specific databases [74]. On the other hand, the gut microbiota community is a set of units that represent an interface between host and a specific nutritional environment. Each unit, depending on space and temporary variations, influences the physiology and metabolism of each host in a different way. The community profiling studies look for the integration of a taxonomic microbial description and metabolites generated by host responses in order to establish therapeutic outcomes aimed at recovering health associated with metabolic diseases [75].

### 3.3. Database for Microbiota Genomic Data

With respect to the databases, some examples are mentioned. KEGG (http://www.kegg.jp/) is an encyclopedia of genes and genomes originally developed in 1995 to define nodes of molecular networks. This platform assigns functional meanings to genes and allows for comparisons with high accuracy. The functional information is stored as a KEGG Orthology (KO) related to specific genes and proteins with a functional background. Also, it develops networks of molecular interactions, reactions, and relations as pathway maps and other representations [76].

Another database is the Human Microbiome Project (HMP), which was launched in 2008 and is a collection of information about the diversity of the human microbiome in search of patterns associated with physiology and disease. Analysis of the taxonomic and metagenomic composition of the microbiome in a considerable healthy cohort is possible. Recently, the second phase of this project, the Integrative Human Microbiome Project (IHMP) Consortium (2013–2016), which is the search for the relationship between the microbiome in human health and disease in models of pregnancy, inflammatory bowel disease, respiratory viral infection, and the onset of type 2 diabetes, was launched. The database was generated using techniques like mass spectrometry, gene expression, and metagenomics for microbial composition in each disease model [77].

Another of the databases is Mechanism of Action of the Human Microbiome (MAHMI), which is based on the European Union Project on Metagenomics of the Human Intestinal Tract (MetaHIT) established in 2007, whose first objective was to create a repertoire of the microbial genes from the intestinal tract and to thus characterize such microbial consortia. MAHMI comprises information from 300 billion sequences related to peptides produced by the human gut microbiome with cell cycle-regulating and immunomodulatory properties based on in silico protein digestion by proteases and global sequence similarity comparisons with MetaHIT [78].

An example of an application of the database is by Davis et al. in 2017, who performed a cross-sectional case–control study with 81 adult participants (mean = 33 years old) from Alabama, USA, that included participants of both genders with different types of diet and body mass index (BMI), whose stool samples were treated for 16S rRNA gene amplification and high-throughput community sequencing data for diversity analysis. In this work, the Bacteroidetes phylum, considered a healthy-gut bacteria, was most prevalent in overweight or obese persons that consumed westernized diets and also in normal-weight persons who consumed such diets. A high association between processed diets and Firmicutes (including *Dialister* sp., *Oscillospira* sp., and others) was seen. The results supported the hypothesis that a Western diet has a larger impact on gut diversity than does BMI, and “healthy persons” with a normal BMI but with bad dietary habits presented with gut dysbiosis [79].

**Figure 1:**
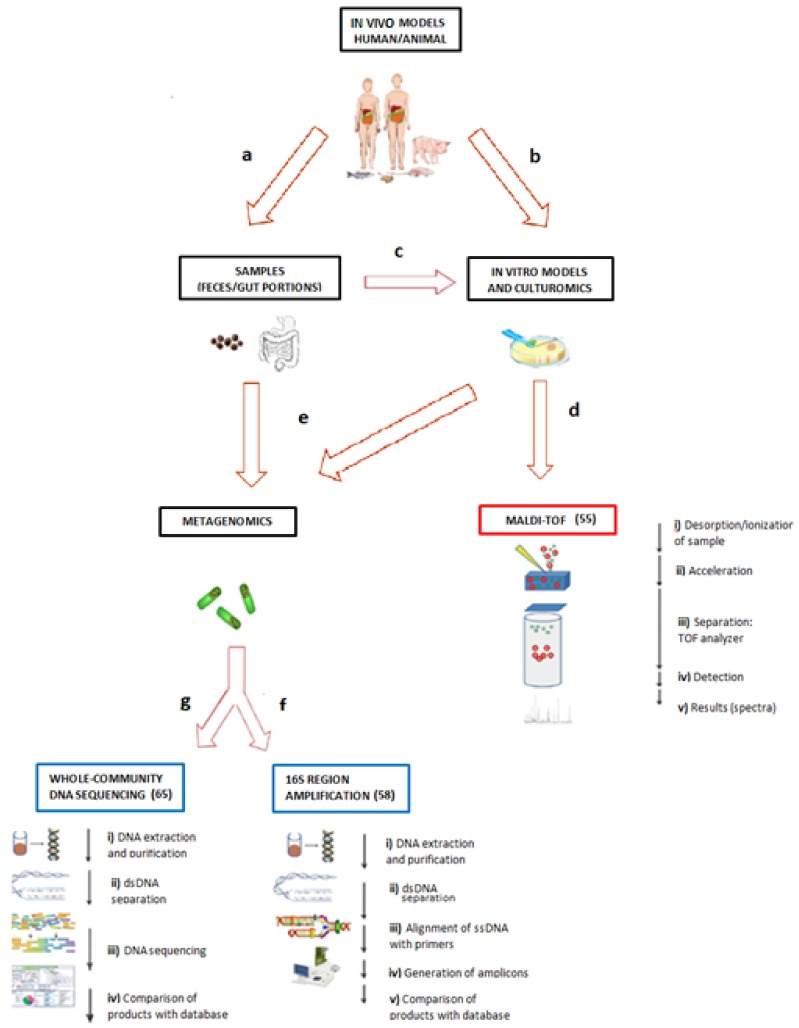
Primary possibilities for microbiota research. To understand the roles and interactions of the microbiota, we can start from the animal sources for (**a**) the development of an in vivo model and to obtain samples such as feces or microbiota from the gut, and (**b**) to obtain gut regions and to develop in vitro continuous organ cultures that mimic the biological environment. Likewise, once the samples from the animal models are obtained, (**c**) culturomics can be used as a powerful approach to identify the uncultured members of the gut, search for differences between species at more than the phylum level, and generate results more quickly by coupling tools such as (**d**) matrix-assisted laser desorption/ionization–time of flight (MALDI–TOF) to generate valid and reproducible results. On the other hand, for both the culture and samples, the researcher can use (**e**) the metagenomics approach, which can be divided into two main techniques: (**f**) The 16S ribosomal sequence amplification, which provides information related to phyla and the abundance in the sample, or (**g**) whole microbial DNA sequencing, which provides more information than simply the phylum or abundance, showing the relationship between microbial enzymes, metabolic pathways, or genetic expression, and diseases such as obesity and others. This figure was made using Creative Commons resources and cannot be copyrighted by others.

In 2017, Shang et al. published a short-term (seven-week) high-fat diet (HFD) study using C57BL/6J mice, where a specific group was subjected to only five weeks of HFD, and the last two weeks they were returned to a low-fat diet in an effort to evaluate changes in the gut microbiota and the effect of HFD cessation. The 16S rRNA from stool samples were sequenced and analyzed against Illumina libraries, and the functional analysis of the bacterial DNA was analyzed against the KEGG database using STAMP software. The return of the mice to low-fat diets from week 5 to 7 resulted in normalization of body weight, levels of blood glucose, and levels of hepatic triglycerides to similar levels compared to those in the control group. However, two weeks of HFD cessation was not enough to restore the composition and diversity of the gut microbiota. On the other hand, lipid, starch, and sucrose metabolic properties analyzed using whole bacterial DNA were significantly lower compared to the HFD group, but diversity and composition of the microbiota were only partially restored [80]. The primary possibilities for microbiota research, in vivo or in vitro models, the origin and treatment of the samples, the main molecular and proteomic methodologies, as well as the most used GEMs and databases for microbiota genomic, are integrated in Figure 1.

## 4. Fecal Microbiota Transplantation as a New Therapeutic Approach for Obesity

The study of the microbiota and its role in the development of obesity and metabolic diseases has opened the possibility of it being considered as a potential treatment for obesity [81] and several other inflammatory diseases [4]. Within the many possibilities that this field represents, fecal microbiota transplantation (FMT) has proven to be a promising alternative. For example, in a study in 2012 by Vrieze et al., gut microbial infusion from the small intestine was transplanted via a gastroduodenal tube from healthy lean donors to obese human subjects that had a metabolic disorder. They found that at six weeks after the allogenic transfer, the recipients showed an improvement in peripheral insulin sensitivity, a trend of improvement in hepatic insulin sensitivity, and an increase in gut microbial diversity and proportion (especially in butyrate-producing bacteria), which all pointed to metabolic improvement [82]. Nevertheless, more clinical trials are needed to evaluate the potential of FMT as an effective therapeutic approach for metabolic diseases. It is important to note the need for standardization of FMT transplant procedures and the measurement of adverse reactions due to possible complications during pretreatment with the infusion, the delivery route, and the methodology [83], since the above-mentioned tools and methodologies may be of great importance for the effectiveness of this therapy

## 5. Concluding Remarks and Perspectives

Obesity remains a critical health problem around the world, which makes it imperative to understand its pathology and to search for new treatments. A large amount of research has been done in the field of gut microbiota and its impact on host metabolism, which has become a promising target for new approaches and solutions, but the relationship between the microbiota and obesity remains an intricate question where new information, as well as new questions, emerge day by day. The reductionist perspectives used in past research are no longer sufficient to adequately or precisely explain how the microbiome modulates host metabolism and physiology.

Technological advances and new applications have helped in the confrontation of this challenge. However, it is still necessary to develop humanized animal models that more accurately resemble the human species and systems that allow the efficient matching of the interaction of the microbes with the host, not only in a portion of the digestive tract, but in a completely integral context. A deeper characterization of the components of the microbiota is also needed since even recent work related to the subject has focused only on the diversity of species, disregarding aspects of great importance such as the specific strain, the involvement of viruses and other agents, as well as the integration of proteomics and metabolomics to outline a more complete picture that may be solved with efforts to generate multidisciplinary teams and a constant updating of new techniques and methods within the scope of research.

Finally, all the knowledge generated by the study of the microbiota opens up possibilities for translational medicine such as fecal transplantation being considered as a possible therapeutic alternative in patients with obesity and metabolic diseases. However, more clinical trials are needed in order to verify the approach as a safe and effective intervention.

## 6. Search Strategy and Selection Criteria

Flow of information through the different phases of this review are presented in supplementary Figure S1. The literature search was made through PubMed for English-language published material from 1 January, 2013, to 22 August, 2018, using a combination of keywords such as *obesity*, *metabolic disease*, *microbiota*, *microbiome*, *animal models*, *microbiota database*, and *experimental models*. From this search, 695 results were obtained, of which 50 were considered to be relevant articles based on their analysis and their inclusion of information directly related to innovative technical approaches or different in vivo models for the study of the relationship between gut microbiota and obesity. After reviewing the information, a second search was performed using the same date range and combination of keywords previously mentioned in combination with new keywords such as *metagenomics*, *culturomics*, *honeybee*, *zebra fish*, *pig*, *hydra*, *rat*, *mice*, and *germ-free*. From this second search, 40 results were considered relevant articles. We excluded articles with repetitive information. Other articles with greater antiquity were included when considered to be classical references. In total, one web page (WHO’s page), two books, 29 reviews, and 51 original articles were cited in this review.

## Figures and Tables

**Table 1:** Main complications in microbiota research using human individuals [5].

Complications in Human Host Models	Solutions
Variation in host genome	To control genotype variations in in vivo animal models To study individual human genome projects To look for interpersonal variations between monozygotic and dizygotic human twins
Environmental exposures (toxins, antibiotics, diet)	All of these can be controlled in in vivo animal models
Tractability	In vivo animal models offer the possibility of examining remote regions of the gut
Difficult-to-replicate experiments due to unique microbiota of each individual	Possibility of transplanting the same microbiota into multiple animal hosts

**Table 2:** Animal models for microbiota research (images from Creative Commons).

Animal Model	Main Characteristics of the Model	Aspect of the Microbiota to Study	Methodology Employed	Reference
(A) Hydra (*Hydra* spp.) 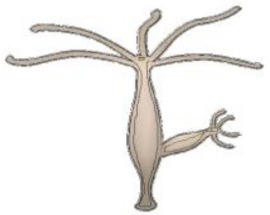	Tube-like body (similar to the human gut) Shares ancestral genes with humans Established protocols for generating germ-free or gnotobiotic animals	Microbe–microbe relationships (including virome) and the impact on the host	In vivo system	[45]
(B) Honeybee (*Apis mellifera*) 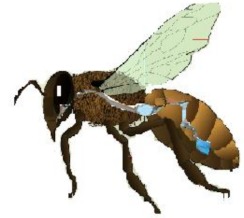	Lower complexity of bacterial diversity All members of the honeybee microbiota can be cultured Established protocols for generating microbiota-free bees and recolonizing bees	Function of bacteria in bee gut species	In vivo system for strain interactions 16s rRNA sequencing	[46]
(C) Zebrafish (*Danio rerio*) 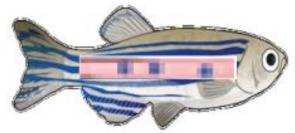	High reproduction rate Environment can be thoroughly sampled Can be raised with the same diet their entire lives	Changes in microbial communities under a constant diet and trough different stages of age Effects of dietary fat on microbiota composition	16s rRNA sequencing	[20]
(D) Mice (*Mus musculus*) 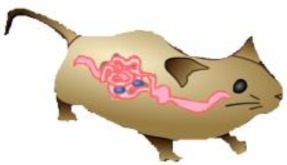	Germ-free mice Small size, large litters, and rapid generation time Techniques for maintaining a sterile environment in GF or gnotobiotic animals are critical	Host–microbe interactions Role of microbiota in homeostasis, health, and diseases Role of the interaction between diet and microbiota and the mechanisms of obesity Effect and mechanisms of inoculation with known microbes	16s rRNA sequencing Metabolomics, identification, and quantitation of metabolites	[47]
(E) Rat (*Rattus novergicus*) 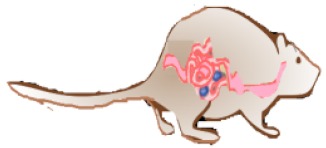	Similar phyla in the gut compared to humans Good models for specific pathogen-free (SPF) experiments	Effect of certain probiotics and prebiotics on the microbiota Effect of diet on the microbiota Role of the microbiota in diseases like obesity	Amplification of bacterial 16S rRNA Microbial metabolites through gas chromatography fitted with a quadrupole mass spectrometry unit	[30]
(F) Pig (*Sus scrofa*) 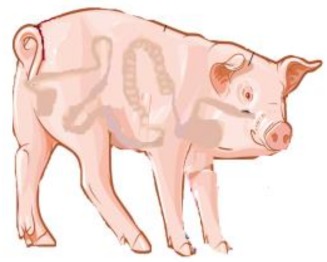	Similarities to humans in gastrointestinal tract functions, anatomical structure, metabolism, nutritional requirements, and bacterial phyla (Bacteroidetes and Firmicutes) As an obesity model, pigs are prone to sedentary behavior and fatten, similar to humans Distribution of fat and adipocyte size are similar in both species	In obesity models, the microbiota interactions can be assessed under more controlled conditions in pigs than in human subjects	qPCR available for amplification and quantification of target bacterial group and total bacteria Analysis of microbial metabolites such as ammonia and short chain fatty acids (SCFAs) using gas chromatography	[48]

**Table 3:** Biases among molecular techniques.

Technique/Process	Biases	Reference
Pyrosequencing	15% of gram-negative bacteria are overlooked compared to transmission electron microscopy (TEM) analysis	[67]
PCR amplification	Bile salts and complex polysaccharides in feces inhibit amplification and affect assay accuracy	[68]
	Some polysaccharides mimic the structure of nucleic acids and affect the enzymes	[69]
Disruption of bacterial membranes	Specific bacterial taxa have differences in cell wall membrane integrity	[70]
DNA extraction methods	Different cell wall disruptors (enzymes, chemical agents, beads) and variables such as exposure time and DNA purification procedures may affect microbiota profiling	[71]

## Supplementary Materials

The following are available online at http://www.mdpi.com/1422-0067/19/12/3827/s1, Figure S1: Flow diagram of search strategy and selection criteria.
