# Supplementary figures and images for: The Human Microbiota and Obesity: A Literature Systematic Review of In Vivo Models and Technical Approaches
Source: Int J Mol Sci. 2018 Nov 30;19(12):3827. doi: 10.3390/ijms19123827 (PMC6320813; doi:10.3390/ijms19123827)

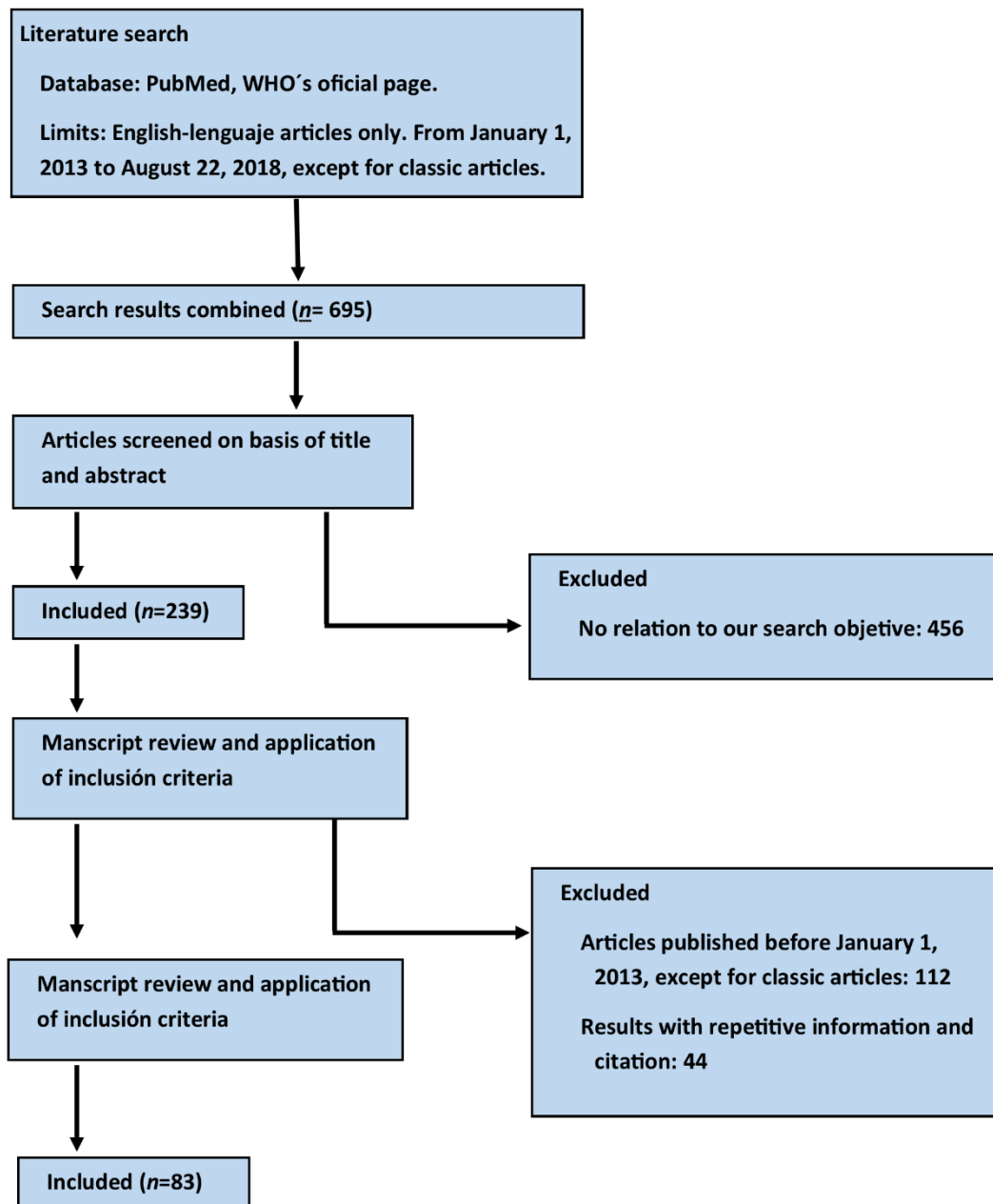

Figure S1. Flow diagram of search strategy and selection criteria.

Supplement: Supplementary file 1 [file ijms-19-03827-s001.pdf]
